# Effect of Health Education on Nutritional Status of Elderly Population in a Rural Area in Delhi: An Interventional Study

**DOI:** 10.7759/cureus.83529

**Published:** 2025-05-05

**Authors:** Jeevan Jyoti Meena, Anshumali Singh, Nidhi Gupta, Gajendra S Meena

**Affiliations:** 1 Community Medicine, Vardhman Mahavir Medical College and Safdarjung Hospital, New Delhi, IND; 2 Community Medicine, Atal Bihari Vajpayee Institute of Medical Sciences and Dr. Ram Manohar Lohia Hospital, New Delhi, IND; 3 Community Medicine, Maulana Azad Medical College, New Delhi, IND

**Keywords:** elderly, health education, mini nutritional assessment, nutrition, rural

## Abstract

Background: The health of the elderly is an important issue defining the health status of the population. Malnutrition significantly increases morbidity and mortality and compromises the outcomes of other underlying conditions and diseases. The malnourished elderly are more likely to require health and social services, have more hospitalizations, and cause a burden on caregivers.

Aim: To study the effect of health education on the nutritional status of elderly aged 60 years and above in a rural area of Delhi.

Methodology: A community-based interventional study was conducted in the Barwala village, which is a rural area in Delhi. A total of 205 elderly were assessed using the Mini Nutritional Assessment (MNA) Tool. Health education was provided as an intervention using poster presentation, audio-visual aids, and pamphlets distribution.

Statistical analysis: Quantitative data was expressed by mean and SD, and tested by Student’s t-test (paired) or Mann-Whitney U test, while qualitative data was expressed in percentage and tested by chi-squared test or Fisher’s exact test.

Results: 83 (40%) subjects were malnourished, and a similar number, i.e., 84 (41%), was at risk of malnutrition according to the MNA score. The prevalence of malnutrition before and after the intervention was 83 (40.5%) and 63 (30.7%), respectively, which showed a 24.1% decrease in the prevalence of malnutrition after the intervention.

Conclusions: There was a high prevalence of malnutrition and risk of malnutrition among the elderly.

## Introduction

The World Health Organization (WHO) has stated that aging populations will present new challenges to healthcare. The health of the elderly is a crucial factor in determining the overall health status of the population. In India, the geriatric age group (aged 60 years and above) constitutes 8.6% of the total population of the elderly in India as per the 2011 census. People aged 60 and older make up 12.3% of the global population, and by 2050, that number will rise to almost 22 per cent [1].

Malnutrition significantly increases morbidity and mortality and compromises the outcomes of other underlying conditions and diseases. It may delay recovery and prolong hospitalization, lead to increased susceptibility to infection, impede individuals’ independence and quality of life, and even increase the risk of death in many patients. Malnutrition poses a huge economic cost to society. The malnourished elderly are more likely to require health and social services, have more hospitalizations, and causes a burden on caregivers [2].

The Mini Nutritional Assessment (MNA) scale was developed to diagnose the risk of malnutrition in elderly people. It provides a simple and quick evaluation of the nutritional state of elderly people in hospitals, geriatric institutions, or the community. It is an 18-item validated nutritional screening instrument that has a sensitivity of 54-90% when compared with a detailed nutritional assessment. It is simple and noninvasive, which facilitates its use in the community. It correlates well with biochemical markers of malnutrition [3].

Socio-economic status and functional ability are often major indicators of nutritional status. The cost of housing and medical expenses (most notably, medication) often competes with the money needed for food. When financial concerns are present, meals are often skipped, and food that is purchased may not provide a nutritionally adequate diet [4].

Declines in functional status, both physical and cognitive, affect a person's ability to shop for food and to prepare meals. Loss of instrumental skills related to activities of daily living (e.g., shopping, transportation, meal preparation, house-keeping, taking medications, managing finances, using the telephone) leads to dependence on others [5].

This study was conducted since only a few studies in India assessed the effect of health education on the nutritional status of the elderly population. The objective of the study was to assess the effect of health education on the nutritional status of the elderly population.

## Materials and methods

It was a community-based longitudinal intervention study conducted in Barwala village situated in Narela Tehsil of the northwest district of the National Capital Territory of Delhi, a rural health training center (RHTC) of the Department of Community Medicine, Maulana Azad Medical College, New Delhi. It was conducted from January 2018 to December 2018. The sample size was calculated using the formula $N = \left( Z_{\alpha/2} + Z_{\beta} \right)^2 \left( \frac{P_1 Q_1 + P_2 Q_2}{(P_1 - P_2)^2} \right)$. Using 20% as the prevalence of malnutrition in the elderly in India, the final sample size came out to be 195 [6]. Taking a 10% allowance for loss to follow up, the final sample size was 215. A total of 205 elderly (five moved out of the study, one participant migrated to another place, and four could not be traced) were included in the final analysis. The elderly population aged 60 and above residing in Barwala village for at least six months was included. Elderly individuals who were severely ill and mentally disoriented were excluded from the study. The primary outcome was the change in nutritional status of the elderly population after intervention. Ethical approval was taken from the Institutional Ethics Committee, Maulana Azad Medical College and Associated Hospital, F. no 17/IEC/MAMC/2017/249.

Sampling technique

**Figure 1:**
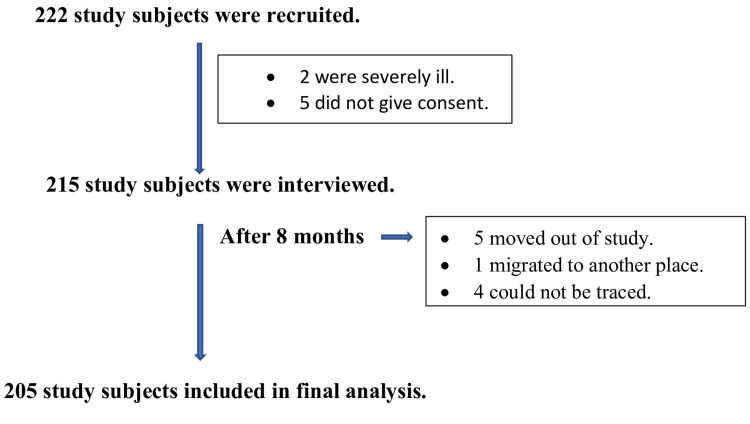
Flow chart depicting recruitment of study subjects

According to a survey conducted in 2017, the total population of Barwala was 5,067. The number of elderly people aged 60 and above was 395. House-wise listing of study subjects was done by house-to-house visit. The number of study subjects were selected by using a simple random sampling method using a simple random number table. The individuals fulfilling the inclusion criteria were selected for the study. All the study subjects meeting the eligibility criteria were recruited for the study. Finally, 205 subjects responded till the end of the study and were included in the final analysis. The details are shown in the flow chart (Figure 1). The existing MNA questionnaire was used to assess the nutritional status of study subjects. This was translated to Hindi and pretested and retranslated back to English. Modified B.G. Prasad scale was used for socio-economic classification of each study subject [7].

Methodology

The study was conducted in three phases.

Pre-intervention Phase

The elderly who agreed to participate in the study were interviewed after getting written informed consent. After recording the preliminary data (socio-demographic details, lifestyle characteristics, somatic factors, functional factors, etc.), the elderly were subjected to the interview and examination through the MNA. It was carried out for two months. If a study subject was not available at the time of first visit, subsequently, two more visits were done. If, even after the third visit, the study subject was not available, then the next eligible elderly person was recruited for the study.

Intervention Phase

Health education material on types of physical activity and their benefits, benefits of eating a balanced and healthy diet, and getting regular health check-ups was prepared and provided in the village in groups of 10 to 12 study subjects using poster presentation and audio-visual aids. Weekly, three sessions, two hours each, were arranged at a convenient place and time in the village. Education materials in the form of pamphlets were distributed after the session. The contents of pamphlets were dos and don’ts regarding proper nutrition and a healthy lifestyle. This phase was carried out for two months. Reinforcement of health education was done after three months using the same technique.

Post-intervention Phase

After a total period of eight months of intervention, post-intervention nutritional assessment was done using the same questionnaire for two months.

Statistical analysis

The collected data was entered in Microsoft Excel and analyzed using SPSS-PC-25 version. Data was expressed in mean ± SD or percentages/proportions. The association between risk factors and malnutrition was tested by chi-squared test or Fisher’s exact test. The difference between means for paired groups was analyzed using the paired t-test or McNemar's test for normally distributed data. A p-value less than 0.05 was considered statistically significant. 

## Results

Out of 205 elderly, 83 (40.5%) were malnourished and 84 (41.0%) were at risk of malnutrition; only 38 (18.5%) were found to have normal nutritional status. The majority of participants across all categories belonged to the 60-70 years age group. Among the malnourished, 41 (72.3%) were in the age group of 60-70 years, 12 (14.4%) were aged 70-80 years, and 11 (13.2%) were aged 80-90 years. Among those at risk of malnutrition, 65 (77.4%) were aged 60-70 years, and among the normal elderly, 26 (68.4%) were in the age group of 60-70 years. 48 (57.8%) and 50 (59.5%) elderly among the malnourished and at-risk-of-malnutrition group, respectively, were females. Among all the groups, i.e., 60 (72.3%) of the malnourished, 68 (81.0%) at risk of malnutrition, and 34 (89.5%) of the normal elderly followed Hinduism. Of the total, the majority, i.e., 73 (35.6%) of study subjects were living in joint families, followed by 52 (25.4%) who lives in nuclear families. In the malnourished group, 49 (59.0%) belonged to joint families and 34 (41.0%) to nuclear families, whereas among those at risk, 51 (60.7%) were from joint families and 33 (39.3%) from nuclear families. 69 (83.1%) of malnourished individuals belonged to Class III-V, and only 14 (16.9%) belonged to Class I-II. Similarly, among those at risk, 68 (81.0%) were from Class III-V and 16 (19.0%) from Class I-II (Table 1).

**Table 1:** Baseline characteristics of population according to the MNA score (N=205) MNA: Mini Nutritional Assessment

Characteristic		Malnourished n (%)	At Risk of Malnutrition n (%)	Normal n (%)
Age	60–70	41 (72.3)	65 (77.4)	26 (68.4)
	70–80	12 (14.4)	8 (9.5)	6 (15.8)
	80–90	11 (13.2)	1 (48.8)	6 (15.8)
	Total	83 (100.0)	84 (100.0)	38 (100.0)
Gender	Male	35 (42.2)	34 (40.5)	1 (47.4)
	Female	48 (57.8)	50 (59.5)	20 (52.6)
	Total	83 (100.0)	84 (100.0)	38 (100.0)
Religion	Hindu	60 (72.3)	68 (81.0)	34 (89.5)
	Muslims	17 (20.5)	13 (15.5)	2 (5.3)
	Others	6 (7.2)	3 (3.6)	2 (5.3)
	Total	83 (100.0)	84 (100.0)	38 (100.0)
Type of Family	Nuclear	34 (41.0)	33 (39.3)	18 (47.4)
	Joint	49 (59.0)	51 (60.7)	20 (52.6)
	Total	83 (100.0)	84 (100.0)	38 (100.0)
Socio-economic Status (using modified BG Prasad Scale)	Class I and II	14 (16.9)	16 (19.0)	4 (10.5)
	Class III, IV and V	69 (83.1)	68 (81.0)	34 (89.5)
	Total	83 (100.0)	84 (100.0)	38 (100.0)

**Figure 2:**
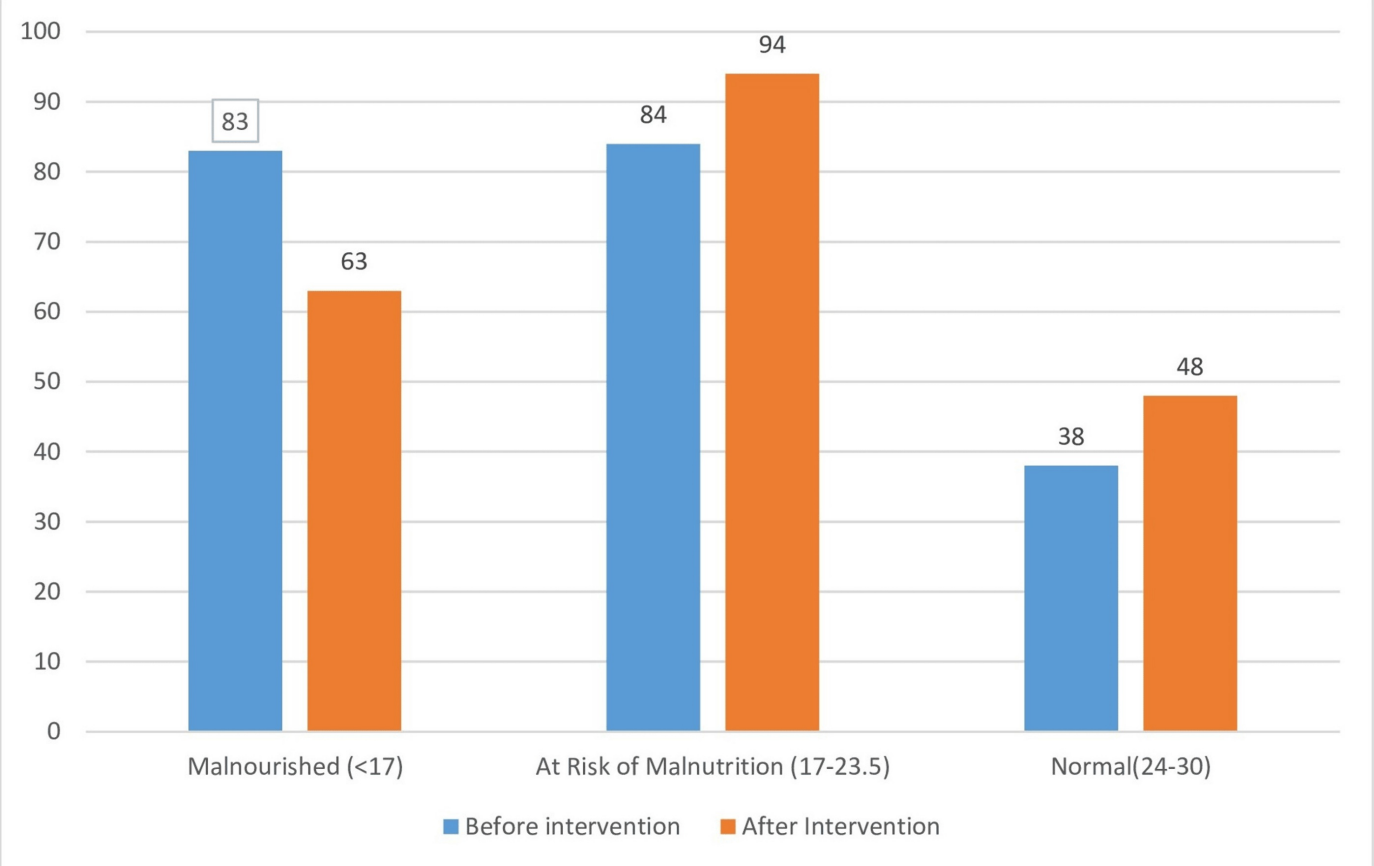
Nutritional status-wise distribution of study subjects based on MNA score pre and post intervention (N=205) MNA: Mini Nutritional Assessment

Post intervention, the number of malnourished elderly reduced to 63 (30.7%) from 83 (40.5%), showing a decrease of 22.9%. Those at risk of malnutrition increased to 94 (45.9%) from 84 (41%) after the intervention. The number of participants with normal nutritional status increased to 48 (23.4%) from 38 (19%) (Figure 2).

On performing a paired t-test, there was a statistically significant improvement post intervention, with the mean score increasing from 17.4 ± 4.6 to 18.6 ± 4.17 as a result of the intervention (p = 0.006). Similarly, there was a statistically significant increase in BMI from a mean of 20.6 ± 2.91 to 21.1 ± 2.56 after the intervention (p < 0.001). The proportion of current smokers slightly decreased from 88 (42.9%) to 86 (42.0%), while the number of past smokers increased slightly from 60 (29.3%) to 62 (30.2%); however, this change was not statistically significant (p = 0.157). Similarly, the proportion of current alcohol users declined from 56 (27.3%) to 54 (26.4%), and past users increased from 35 (17.1%) to 37 (18.0%), but the change was not significant (p = 0.157) Those exercising for one to three days per week rose from 114 (55.6%) to 119 (58.0%), and those exercising for more than three days per week increased from 61 (29.8%) to 66 (32.2%). Meanwhile, the number of participants who did not engage in any exercise decreased from 30 (14.6%) to 20 (9.8%). On performing McNemar's test, there was a statistically significant association between exercise done by the subjects before and after intervention (p = 0.03) (Table 2).

**Table 2:** Pre and post intervention scores and distribution of study subjects (N=205) ^*^: p < 0.05 is considered as significant; ^#^: paired t-test; ^$^: McNemar's test

Parameter	Score		Before intervention n (%)	After Intervention n (%)	p value
Nutritional status, mean score ± SD			17.4 ± 4.6	18.6 ± 4.17	0.006^*#^
BMI (mean ± SD)			20.6 ±2.91	21.1 ±2.56	<0.001^*#^
Lifestyle characteristics					
Smoking		Current	88 (42.9)	86 (42.0)	0.157*
		Never	57 (27.8)	57 (27.8)	
		Past	60 (29.3)	62 (30.2)	
Alcohol		Current	56 (27.3)	54 (26.4)	0.157*
		Never	114 (55.6)	114 (55.6)	
		Past	35 (17.1)	37 (18.0)	
Exercise done for at least 30 min/ day		1-3 days	114 (55.6)	119 (58.0)	0.03^*$^
		>3 days	61 (29.8)	66 (32.2)	
		Not done	30 (14.6)	20 (9.8%)	

## Discussion

Socio-demographic factors

In the present study, there was no significant statistical association between malnutrition with socio-demographic characteristics and socio-economic status, but Lahiri et al. had reported that females (59.4%) were significantly more malnourished than males (40.6%) [8]. Joymati et al. and Ghimire et al. also reported that females were more malnourished than males and malnutrition was significantly associated with higher age and female gender [9,10]. Other studies conducted by Kansal et al. and Bishnoi et al. reported no significant statistical association between malnutrition with socio-demographic characteristics, and this finding is similar to that of the present study [11,12]. This could be attributed to traditional habits of eating after children and the practice of sharing among family members, which could result in malnutrition. Ramya et al. reported that nutritional status was found to worsen significantly with advancing age, which is contrary to the present study, which could be due to the smaller number of elderly in advanced age in the present study [13]. Singh et al. reported that males were more malnourished than females, which could be due to a higher proportion of males (62%) in that community, which is contrary to the present study [14].

Change in nutritional status after health education in study subjects

A pre-post study by Ming et al. in Hong Kong in 2013 on the effectiveness of a food education program in improving the appetite and nutritional status of elderly adults living at home showed a 25% decrease in malnourished elderly, which is similar to the present study, but the health education given was different from the present study [15].

Ghasemi et al. showed that 35.9% of the elderly had very severe and severe malnutrition prior to the study, decreasing to 18.3% following training, which is lesser than the present study, as the mode of health education was different between the two studies [16]. 

Strengths and limitations

A validated questionnaire was used to elicit the prevalence of malnutrition. Audio-visual aids with the help of a tablet, in addition to pamphlets, as a part of health education, helped in better understanding of malnutrition. The limitations of the study were that number of meals was taken as a measure of malnutrition as per the MNA questionnaire and not the calorie consumption. Data collection was based on history, which might have under- or over-reported the risk factors.

## Conclusions

There was a high prevalence of malnutrition and those at risk of malnutrition among the elderly. It is important to prevent malnutrition in the elderly and identify those at risk of malnutrition. This may be done by screening elderly people, which should be done periodically with simple measures such as the MNA. Treatment of malnutrition should be multifactorial. Health education may be given to primary caregivers who can help in identifying the elderly at risk. Awareness can be created among family members about the importance of early detection of malnutrition. Health education activities can be done using pamphlets that can help in a better understanding of malnutrition.

Screening of the elderly should be done with simple measures such as MNA for early detection of malnutrition. Similarly, mid-upper arm circumference and calf circumference could be used to detect malnutrition. Healthcare providers should provide health education on malnutrition and nutritional support to the elderly and family members.
